# Emergence of complex behavior in pili-based motility in early stages of *P. aeruginosa* surface adaptation

**DOI:** 10.1038/srep45467

**Published:** 2017-04-10

**Authors:** Yifat Brill-Karniely, Fan Jin, Gerard C. L. Wong, Daan Frenkel, Jure Dobnikar

**Affiliations:** 1 Department of Chemistry, University of Cambridge, Lensfield Road, CB2 1EW, Cambridge, UK; 2 Institute for Drug Research, Faculty of Medicine, The Hebrew University of Jerusalem, Jerusalem, 91120, Israel; 3 Hefei National Laboratory for Physical Sciences at Microscale, Department of Polymer Science and Engineering, CAS Key Laboratory of Soft Matter Chemistry, University of Science and Technology of China, Hefei 230026, P. R. China; 4 Bioengineering Department, Chemistry and Biochemistry Department, California Nano Systems Institute, University of California, Los Angeles, CA 90095, USA; 5 Beijing national laboratory for condensed matter physics & CAS key laboratory of soft matter physics, Institute of Physics, Chinese Academy of Sciences, Beijing 100190, China; 6 School of physical sciences, University of Chinese Academy of Sciences, Beijing 100049, China

## Abstract

*Pseudomonas aeruginosa* move across surfaces by using multiple Type IV Pili (TFP), motorized appendages capable of force generation via linear extension/retraction cycles, to generate surface motions collectively known as twitching motility. *Pseudomonas* cells arrive at a surface with low levels of piliation and TFP activity, which both progressively increase as the cells sense the presence of a surface. At present, it is not clear how twitching motility emerges from these initial minimal conditions. Here, we build a simple model for TFP-driven surface motility without complications from viscous and solid friction on surfaces. We discover the unanticipated structural requirement that TFP motors need to have a minimal amount of effective angular rigidity in order for cells to perform the various classes of experimentally-observed motions. Moreover, a surprisingly small number of TFP are needed to recapitulate movement signatures associated with twitching: Two TFP can already produce movements reminiscent of recently observed slingshot type motion. Interestingly, jerky slingshot motions characteristic of twitching motility comprise the transition region between different types of observed crawling behavior in the dynamical phase diagram, such as self-trapped localized motion, 2-D diffusive exploration, and super-diffusive persistent motion.

Type IV Pili (TFP) are nanomotor/motility appendages capable of generating forces in the 100 pN range. These filaments, which function via extension-attachment-retraction cycles, are the strongest linear molecular motors known to date^1^,^2^,^3^,^4^. TFP are responsible for a rich diversity of bacterial phenomena. *P. aeruginosa* translocate across surfaces by deploying multiple TFP, resulting in complex motions collectively known as twitching motility^5^,^6^,^7^,^8^,^9^,^10^,^11^,^12^,^13^. TFP also play key roles in bacteria-bacteria interactions and bacterial-surface interactions important to pathogenesis^14^,^15^,^16^,^17^,^18^,^19^,^20^,^21^,^22^,^23^,^24^,^25^,^26^. TFP driven twitching motility is complex, and can be influenced by many factors, including substrate topography, fluidity and stiffness, and also by surface coating, secretion of biological molecules and oxygen levels^27^,^28^,^29^,^30^,^31^,^32^. Recent work on *Neisseria gonorrhoeae* suggests that multiple TFP nanomotors can collectively function via a tug-of-war type mechanism^33^. Moreover, it was shown that *Neisseria gonorrhoeae* transition between fast and slow motion modes, triggered by oxygen levels and proton motive force^32^,^34^,^35^. In *P. aeruginosa*. the interactions between TFP and bacterial exopolysaccharides deposited on the surface can guide the organization of microcolonies^36^.

Analysis of high speed movies of *P. aeruginosa* reveals that they switch between fast and slow modes, where the fast modes are relatively rare, of short duration, and associated with rotations of the cell (“slingshot”)^37^. In similar systems it was also shown that within the same population of *P. aeruginosa*. other distinct dynamical behaviors were observed: while some of the bacteria were consistently performing a super-diffusion motion, others were trapped with a sub-diffusion motility^16^,^17^. The relations between these movements and the slingshot-like motion in *P. aeruginosa* are not known at present.

One poorly understood phenomenon is the emergence of complex twitching motility itself in early surface attached bacterial cells. *Pseudomonas* cells arrive at a surface with low levels of piliation and TFP activity, which both progressively increase as cells sense the presence of a surface^24^,^25^,^38^,^39^. How do motility phenomena emerge from these minimal under-piliated conditions, and what are the associated requirements on the TFP nanomotors themselves? The problem is compounded by the general lack of systematic experimental data. Although direct observations of TFP have been made in pioneering experiments using Fluorescence Microscopy by Skerker and Berg in 2001^5^, and later by Zaburdaev *et al*.^30^, at present we do not have the full dynamical history of every TFP in a given cell, which makes quantitative analysis difficult.

Here, we establish a multiscale mathematical model of twitching motility bridging the scales from single pilus dynamics and the coordination of the molecular motors to macroscopic properties of bacterial trajectories. We compare the model predictions to experimental trajectories for single cells on glass surface. We do not consider the complications from viscous and solid friction on surfaces in order to asses the minimum degree of complexity needed to reproduce the characteristic motility modes and the slingshot-like jumps observed in the experiments. We find that the main order parameters controlling the type of motility are the average number of surface bound pili and the angular spread of TFP orientation. As schematically displayed in the dynamic phase diagram in Fig. 1, a polarized TFP distribution results in persistent motion, while a wide-spread distribution can lead to persistent, diffusive or trapped behavior. These qualitative observations are robust, although the exact location of the boundaries between the different modes is likely to depend on the properties of the surface, especially in case of nonlinear friction effects such as shear thinning.

Slingshot events in the simulations are fast and occur during a small fraction of total time engaged in surface motility (in agreement with experiments). Interestingly, the range of parameters where they are observed (marked green in Fig. 1), is close to the intersection of the three observed crawling modes. This suggests that the slingshots - in addition to facilitating efficient motion on EPS covered surfaces - play an unanticipated important role in mediating changes in motility strategies in response to environmental conditions.

We found that the slingshot jumps can emerge with as little as two TFP bound to the surface, when they are nearly anti-parallel and under high tension (“high-tension antiparallel configuration”, HTAPC) and one of them is suddenly released. With more than two bound pili the motion becomes less jerky, which is consistent with recent findings of Maier *et al*.^31^,^33^, where they modified number of pili on bacteria and observed increased correlation times with increased pili number. However, even in configurations with two bound TFP, not all the sudden jumps in the model resemble the slingshots observed in experiments where the sudden change in speed is correlated with the angular reorientation: we found such correlation only when TFP were very flexible for rotation around the point where they emanate from the surface of the cell. This suggests that the angular flexibility of the motor is a crucial microscopic feature enabling the *P. aeruginosa* slingshot behaviour.

## Model

We designed a multiscale model of *P. aeruginosa* twitching motility linking the microscopic pili-based mechanisms with the macroscopic properties of bacterial trajectories. The cell is treated as a rigid rod-like body with 12 pili (the number is chosen in accordance with available experiments^5^,^40^) emanating from one of its poles^5^,^40^,^41^,^42^. The pili point to various directions, with equilibrium angles *ϕ*_eq_ that are randomly chosen from a Gaussian distribution with variance *ν*: 
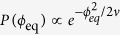
. TFP length changes in time: it is governed by the action of the molecular motors that add or remove pilin monomers resulting in TFP elongation or retraction. The pili are attached to the motor at the cell surface via a flexible hook, while their other end is either free or bound to the surface in a non-specific way (Fig. 2a). In the experiments^3^, it was established that surface-bound pili predominantly retract - bound pili polymerization was observed only under high tension. However, free pili were observed to equally elongate and retract^5^,^34^,^43^. In line with these observations, we assume that each pilus can be in one of the two modes and that the probability of switching between the elongation and retraction depends on the tension in the filament, which is schematically illustrated in Fig. 2b and detailed in the Methods section. The free pili retract or elongate with a rate 0.8 *μ*m/s based on Skerker and Berg^5^ and the bound TFP retract with a rate 0.7 *μ*m/s^34^. Here, we assume that this is also their elongation rate. TFP can shrink to zero length and disappear, however, the total number of pili is conserved and such events are followed by a “birth” of a new pilus in a random direction chosen from the distribution (in another system, of *N. gonorrhoeae*, it was suggested that there is orientational memory in new pili distribution for explaining directional twitching of bacteria that are assumed to be of spherical symmetry).

TFP are modelled as elastic filaments (Fig. 2a). The elastic energy of a filament with a temporary equilibrium length *L*_eq_(*t*) and actual length *L*_p_(*t*) oriented in a direction *ϕ* relative to the cell axes is


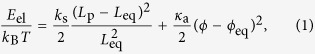


where *k*_s_ = *κ*_s_*L*_eq_/*k*_B_*T* is the linear spring constant that can be estimated from the persistence length of TFP measured in experiments^2^,^5^: *κ*_s_ = 10 *μN*. The filaments bending flexibility was not explicitly considered but is effectively incorporated in the free TFP length. Since bound pili are typically under high tension, significantly bent configurations are unlikely. *ϕ*_eq_ is the orientation of the filament at birth, and *k*_a_ the angular spring constant measuring the effective flexibility for the filaments to deviate from *ϕ*_eq_. The values of the effective rotational flexibility *k*_a_, as well as the variance of pili distribution *ν* have not been determined experimentally and are treated as free parameter in our model.

Bacterial trajectories are simulated using the Kinetic Monte Carlo algorithm (KMC)^44^ considering the following possible processes at each time step (illustrated in Fig. 2a): TFP elongation, retraction, switching between the two, pili rotation, attachment to and release from the surface, and creation of a new pilus (correlated with annihilation of an existing one). At each simulation step, the rate constant for each process is determined and a process is selected with probability proportional to it. The sum of all rate constants determines the lifetime of the present state and thereby the simulation time step^44^. The chosen processes in the KMC step typically result in stretching or compression of surface bound TFP. After each such process we perform Molecular Dynamics based equilibration of the cell position and orientation in order to minimize the tension created by performing the KMC step. In this way we associate single TFP processes with the motion of the cell and introduce cooperativity among TFP. For each set of the parameter values we run several thousands of single-cell trajectories, 1100 seconds long, and average the results collecting data after 100 seconds. These computer-generated trajectories are then compared to the experimental ones - focusing on statistical properties such as the shape of bacterial speed distribution *P*(*u*/u*) and trajectories’ angular persistence.

## Results and Discussion

We performed experiments using advanced tracking methods with a time resolution down to 0.1 s. In the numerical simulations we performed a similar number of simulation runs with the same resolution. The details of the experimental and modeling approaches are described in the Materials and Methods section. Quantitative comparison of the results enables us to determine the values of physical parameters that are difficult to assess by direct experiments and apply the additional insight obtained through the modeling to elucidate the microscopic mechanisms of motility.

### Slingshot bacteria preform two-legged motility

The motility at large is most notably affected by the number of pili that are typically bound to the surface at the same time. From each simulated trajectory we can define the average such number, 〈*N*_B_〉. The value 〈*N*_B_〉 ≈ 1 describes a one-leg motility where a single motor action is responsible for cell propulsion, while for larger values 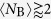, the propulsion is a result of coordination between the motors pulling on pili and is frequently oppressed due to emergence of tug-of-war configurations. Bacteria speed distribution is thus - not surprisingly - qualitatively different for bacteria with different 〈*N*_B_〉. The absolute speed of bacteria in experiment and simulations differs mainly due to the simplicity of the model, e.g. not considering the surface friction. However, by rescaling the speed with the most probable non-zero speed u^*^, it is still possible to quantitatively compare simlations and experiments. Figure 3a shows three distributions of the rescalled speed, *P*(*u*/u^*^), from the simulations (black) compared to the experimental data (orange). A measure for the discrepancies between the model and experiments, 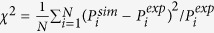, where *N* is the number of bins used for comparison, is plotted in Fig. 3b showing a clear minimum at 〈*N*_B_〉 ≈ 2. Therefore, the best matching trajectories are those where the cells mostly perform two-legged crawling. Figure 3a demonstrates that in case of roughly one-legged crawling, the TFP are often unattached exerting no pulling force on the cell, which results in frequent resting periods and a pronounced peak at zero speed. For higher values of 〈*N*_*B*_〉 there can be frustration due to several bound TFP resulting in trapped configurations with small steps and slow localized speed. Also, in this case TFP length is short (as explained below), resulting in small steps and overall low speed of motion.

Since the rates of TFP elongation and retraction depend on whether they are bound to the surface or not, the order parameter 〈*N*_B_〉 also controls the average length of pili filaments for a given trajectory, as depicted in Fig. 3c. The average length for optimal conditions with 〈*N*_B_〉 ≈ 2 is in sub-micron range, which is not in contradiction with the available experimental observations^5^,^41^.

The number of bound pili 〈*N*_B_〉 depends predominantly on the rates of TFP surface attachment *K*_Ad_ and release *K*_Rel_, therefore on the chemical properties of pili and surfaces. We have scanned a large parameter space of the *K*_Rel_ and *K*_Ad_ values (14 orders of magnitude of their ratio) and and found that everal combinations of these rates can result in a given 〈*N*_B_〉 with an interesting feedback mechanism that results in oscillatory dependence (see Figure S1 on the Online Supplementary Information for a detailed discussion). Comparing simulations and experiments, the best matching speed distribution was obtained with a combination of values: *K*_Rel_ = 130 s^−1^ and *K*_Ad_ = 5 s^−1^. These values were used in the rest of the text unless stated otherwise. However, the physical interpretation of these numerical values is difficult since the friction has not been explicitely considered within our model.

### Role of tension: slingshot mechanism

Jin *et al*.^37^ showed that in the sling-shot motion bacteria change between slow and fast modes, and that while the slow motion is directional, the fast mode (jumps) is often associated with substantial rotation. After obtaining long-time spatially resolved trajectories from both, the experiments and the simulations, we decomposed the trajectories into slow and fast modes of motion in a similar manner as has been done in ref. 37. Jumps are rare events, however, due to the substantial change of the cell position and direction during a jump, they make an important impact on the overall motion. In the simulations we typically observe clearly distinguishable slow and fast modes with an order of magnitude difference in the speed (Fig. 4). As assumed in ref. 37, the fast modes are correlated with the release of a bound pilus. However, only the release events associated with high tension in the filaments lead to fast jumps.

The tension-mediated cooperative action of TFP is illustrated in Fig. 5 where crawling sequences were isolated from the full trajectories. Each point on the diagram represents a translocation event in the simulation. The instantaneous speed *u* during the translocation is plotted against the tension in the filaments *F*/*k*_s_. The distinct types of two-legged motion are illustrated above: if picked at random, a likely configuration is that on the left-hand side with two bound TFP pointing into different directions. In such a state the two bound TFP retract by depolymerization which can potentially increase their tension. Assuming that pili cell-attachments are flexible, allowing for free rotation of TFP around them (see Fig. 6), the accompanying stress in the filaments can be relaxed by rotating relative to the cell axis, which results in slow and persistent motion of the cell with relatively small tension in the filaments. Bacteria spend most of the time in this mode. If a pilus is released in such a state, no jump is observed, since there is not enough tension in the filaments. If, however, the cell persists long enough in the slow mode, it moves down the lower branch of points on the speed-tension plot on Fig. 5, reaching an almost antiparallel pili configuration, and then it becomes difficult to relax the tension in the filaments by re-orientation - as the TFP are now puling into opposite directions. This (at the right-hand side of the lower branch) is the so-called high tension antiparallel configuration, HTAPC, indicative of the obstructed motion phase. At this stage, the probability for a pilus release increases - leading to the fast jump, i.e. hopping to the upper branch of the plot in Fig. 5.

### Flexibility-driven twitching

After a jump, the cell axis is oriented approximately in the direction of the remaining bound pilus. Further on, we argue that the reorientation crucially depends on the two key parameters of our study: the rotational flexibility of the motors, *k*_a_, and the variance of the distribution of pili equilibrium directions, *ν*. The evidence for this claim, as provided by simulations, is presented in Fig. 6a where the frequency of jump orientation is plotted as a function of the angular flexibility *k*_a_ at three different values of the variance *ν*. The magnitude of reorientation during the sling shot is given by the angle *θ* between the cell axis and velocity, as in Jin *et al*.^37^ (Fig. 6b). In case of very stiff attachment, the angles are more or less uniformly distributed (see large *k*_a_ values in Fig. 6a) regardless of *ν*. This is most intuitive as in the case of rotationally stiff attachments high tension could develop in configurations at which the two bound TFP are not necessarily anti parallel, leading to wide distribution of jump angles. For flexible-enough rotations we either observed forward facing jumps with angles around *θ* = 0 for small values of the variance *ν* (left), jumps associated with *π*/2 reorientation (middle) in case of moderately distributed angles, or uniform distribution when *ν* is large (right). Figure S2(a–c) on the Supplementary Information illustrates typical relocation pathways of bacteria with flexible TFP attachment that lead to the above reorientations. In Fig. 6c a comparison to the experiments is shown for selected values of *ν* and *k*_a_ in which the slingshot phenomenon is nicely observed with *π*/2 reorientation. The discrepancy between the experimental and theoretical distribution of jump reorientation is shown in Fig. 6d, at a fixed value of *ν* and varying angular elasticity *k*_a_, and vice versa. It is evident that only in a narrow regime of parameters, i.e. *k*_a_ ≤ 10^−9^ and *π*/8 ≤ *ν* ≤ *π*/3, we observe a quantitative agreement between the experiments and simulations at which slingshot occurs. We also found the best correlation between experiments and simulations in this parameter regime when examining reorientation in the slow motion mode, see Figure S3 in the Supplementary Information. Our findings thus indicate that the angular stiffness must be orders of magnitude smaller than the elastic modulus *k*_s_ of the TFP filaments in order to enable slingshot jumps with roughly *π*/2 reorientation.

The reduced angular stiffness in our model can be related to a Young modulus *E* of the material from which the attachment structure is made of: *E* = *k*_a_*k*_B_*T*/*L*^3^, where *k*_B_*T* is the thermal energy and *L* the relevant length scale. Values *k*_a_ ≤ 10^−9^ translate to elastic moduli below 1 Pa. This is an unusually small value: For comparison, the elastic moduli of rods made of common flexible materials such as protein fibrils, rubber or bamboo are typically between M Pa and G Pa. However, the fact that the TFP apparatus is anchored on two deformable fluid membranes^45^ may allow them to exhibit effectively more angular flexibility than that expected from a consideration of only geometry and elasticity of the cellular attachment structure.

Interestingly, we found that the role of motor flexibility does not end in allowing for tension relaxation by reorientation of TFP and approaching HTAPC. Rather, it also controls the orientation of the cell relative to the anti-parallel pili axis just before a jump event, whereby governing the angle *θ*. The additional insight obtained from modeling allows us to discuss this claim in more detail. Once a cell is stuck in the unfavourable HTAPC, the opposing forces are balanced resulting in vanishing net torque on the cell. At this point rotation of the cell body relative to the bound TFP axis becomes governed by the elasticity: the cell acts like an overdamped harmonic pendulum oscillating around the pili motor positions with a frequency 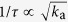 − until a pilus is released from the surface and the cell reorients into the direction of the remaining bound TFP. The time scale *τ* should be compared to the mean lifetime of bound TFP, *τ*_*B*_, which depends on the detachment probability. At *k*_a_ ≈ 10^−9^, our simulations show that *τ* ≈ 1 s and *τ*_*B*_ ≈ 0.05 s. For larger values of *k*_a_, the two time scales become comparable. Therefore, in the flexible case the jump reorientation is practical identical to the angle with which the cell entered the obstructed phase, while for orientationally stiff motors the cell has enough time to reorient and vary its angle relative to the TFP axis within the obstructed phase (see Figure S2(d) on the Online Supplementary Information). The exact value of the crossover angular elasticity 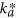 further depends on effects not considered here, *e.g.* friction between the cells and surfaces due to nonspecific interactions. Still, our conclusions should qualitatively remain correct.

Another observation to note is that the initial angle with which the cell enters the obstructed phase, depends on the variance *ν*: if the motors are broadly distributed on the surface of the cell, the initial angles are already smeared out and the jump reorientation angles will be uniformly distributed no matter how flexible the motors are. On the other hand, if the pili angles are very focused, a typical jump will have a forward direction, *θ* ≈ 0 (see Fig. 2 in the Online Supporting Information). It is only in the intermediate case (roughly, *π*/6 ≤ *ν* ≤ *π*/3) that we did observe sling shots with about *π*/2 reorientation.

The above discussion is focused on the typical case with two bound pili. When more than two bind the surface, due to geometrical restrictions, the efficiency of rotation in relaxing the tension becomes limited and the tension in the filaments builds up faster. As a result, highly stretched configurations are observed in a large span of pili orientations and the jumps are not associated predominantly with *π*/2 re-orientations (regardless of *k*_a_ and *ν*), as shown in Fig. 7.

### Crawling modes

**Figure 1:**
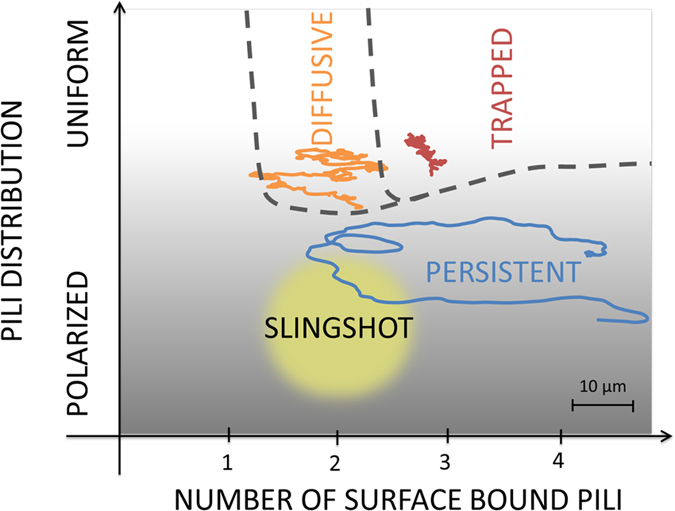
Dynamic phase diagram of twitching motility. Motility regimes observed at different values of the mean number of surface-bound pili and the angular spread of pili from the cell surface: *Diffusive exploration* with small number of bound pili pointing into different directions; *Trapped localized motion* with many bound pili stuck in a tug-of-war situation; and *Directionally persistent trajectories* with TFP localized at the cell poles and mostly pointing into the same direction. Typical simulation trajectories displayed are 1000 s long and the scale bar is 10 *μ*m. The region where the sling shot jumps are emerging is marked with green.

Based on the above knowledge regarding the high flexibility of TFP attachment and the important role of pili tension, in this section we zoom out from the specific slingshot phenomenon to a wide scan of parameter space for exploring general features of the twitching motion. We systematically explored the characteristics of the single cell motility upon variation of 〈*N*_B_〉 (by changing *K*_Ad_ and *K*_Rel_) and *ν*. In Fig. 1 we show a schematic dynamic phase diagram, which is based on several thousand simulation trajectories where we evaluated the mean square displacement (MSD) with the same protocol as in recent experiments^16^,^17^. We observe three distinct distinct types of trajectories: diffusive, trapped and persistent. The super-diffusive motion is defined here by MSD power larger than 1.1, in the diffusive phase the power is 1 ± 0.03, and the trapped (sub-diffusive mode) is with power less than 0.9. The MSD curves are shown in the Supplementary information (Figure S5), where we also list the exponents and the relevant simulation parameters (Table S1).

Not surprisingly, a very localized distribution of TFP angles results in a persistent super-diffusive crawling mode, as the bound TFP pull the cell forward in a cooperative manner, while at a larger values of the spread *ν* the type of motion depends on 〈*N*_B_〉: Trajectories with 〈*N*_B_〉 larger than two are mostly trapped due to the opposing forces pulling in different directions. The diffusive motion appears in a relatively narrow range 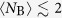 and is governed by single TFP retractions in arbitrary directions. Further decrease to 〈*N*_B_〉 ≈ 1 leads to super diffusive motion due to pili reorientation in the direction of the cell axis causing an effective narrowing of the angular distribution (see Figure S4 of the Supplementary Information for more details). The motion in this case is directional but slow due to the high frequency of periods with no attached TFP.

From Fig. 1 we see that bacteria with sufficiently broad angular distribution of TFP (delocalized distribution of molecular motors) can exchange between three motion phases by altering the number of bound pili - indicating a potential ability to adapt the surface exploration strategy according to their needs (see Figure S4 in the Supporting Information). On the other hand, hyper-piliated bacteria with 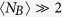 could trigger the transition from super- to sub-diffusive mode by delocalizing the molecular motors - thereby increasing the angular spread of TFP. It is interesting to observe that also the slingshot jumps, despite being rare, play a role in enabling bacteria to switch between the motion phases. The slingshots are observed in the persistent part of the phase diagram with 〈*N*_B_〉 ≈ 2 and *ν* ≈ *π*/5, which is just below the crossroads between the crawling modes (green region in Fig. 1) and emerge from the antiparallel configuration (HTAPC), which effectively broadens the angular TFP distribution (see S6 in the online Supporting Information). Therefore, the slingshots are correlated with switching from persistent to diffusive or trapped motility modes.

## Conclusions

We have designed a coarse-grained model to simulate early stage TFP motility of *P. aeruginosa* on surfaces and quantitatively compared the model predictions to the experiments performed on the cells tracked by high-speed video microscopy. Full quantitative comparison is not the goal since we do not account for interactions with EPS or solid friction. However, our single-pilus-resolved model allowed us to see how complex motility behavior reminiscent of twitching can result from a remarkably small number (as little as 2) of pili simultaneously bound to the surface. We argue that this condition is commonly met in the early stages of bacterial surface adaptation when the cells are underpiliated. Thus twitching bacteria should emerge from the group once single cells within a group exhibit two or more attached TFP. In later stages of adaptation, with multiple pili per cell, such a scenario can still occur if individual TFP attach weakly to the surface - similar to the low-affinity, high-avidity mechanism of surface attachment that has recently been proposed by Dutcher *et al*.^12^ as key to mediating bacterial surface interactions.

The elastic properties of the TFP filaments are key to understanding their collective dynamics^12^. In our model the slingshot jumps only appear if TFP are able to rotate almost freely around their attachment points on the cells indicating that the elastic penalty for stretching TFP has to be several orders of magnitude larger than that for their rotation. Such effective angular flexibility in the coarse-grained model could be explained either by assuming lateral mobility or sufficient structural flexibility of the molecular motors. Recent detailed analysis of the molecular structure of the motor^45^ suggests that the motors are unlikely to be laterally mobile, while the presence of flexible linkers in the structure^45^ makes the assumption of the structural flexibility giving rise to the emergent slingshots plausible. This unanticipated role of flexibility is reminiscent of the buckling instability of the flagellar hook that has been proposed as the key mechanism for bacterial tumbling^46^,^47^.

The dynamical phase diagram in Fig. 1 is helpful for visualizing the relations between different motility behaviors in early stage of *P. aeruginosa* surface adaptation. Our findings point to the ability of bacteria to switch between surface exploration modes by tuning the distribution/orientation of molecular motors on the cell or the number of surface-attached TFP. We predict that a large number of attached TFP will result in a predominantly sub-diffusive motion - qualitatively consistent with previous studies of Deziel *et al*.^48^ who found that hyper-piliated mutants exhibit trapped motion. Moreover, as already argued by Weiss^40^, bacteria may naturally be able to change the number of pili in order to adapt to environmental conditions. The ability to tune the exploration strategy on a single cell level should naturally result in differentiation of bacterial colonies. Indeed, subpopulations exhibiting sub- and super-diffusive behavior have been observed in *P. aeruginosa* colonies^16^,^17^,^28^. In those experiments, we expect behavior to be influenced by secreted exopoplysaccharides such as Pel and Psl. However, it is interesting that we can see both of these regimes in the phase diagram of a minimal system without the effect of EPS.

## Materials and Methods

### Experiments

Flow cells (Denmark Technical University) used here were prepared and sterilized using a standard protocol. After injection of bacterial cultures, the flow cell was typically placed for 15 mins, allowing the attaching of cells to coverslip, afterward, unattached cells were washed out. These surface associated cells were further cultured at 30.0 ± 0.1 °C by flowing (3.0 ml/h) FAB mediums. Meanwhile, an inverted microscope (Olympus IX81) equipped with a 100× oil objective was applied to continuously monitor the surface motility of single cells. The dataset, typically containing 20,000 bright-field images, was acquired within 10 fps in first 6 hrs using a sCMOS camera (Andor Neo). These experiments were repeated multiple times in each identical conditions, thus over 100 datasets (>2 · 10^6^ images) were acquired and analyzed in total, in which the 1800 × 1800 16-bit greyscale images were firstly converted to binary images for detection of bacterial cell with a standard image processing algorithm coding by MATLAB, and the *x* − *y* positions of cell centroids were subsequently determined and linked individually over time by using an efficient particle tracking algorithm we recently developed^16^,^17^,^37^. As already observed in ref. 37, the motion can be categorized into two modes: the slow persistent mode with the mean speed below 0.07 *μ*m/s and the fast mode associated to sudden change of the direction.

### Modeling tension-dependent rate constants

**Figure 2:**
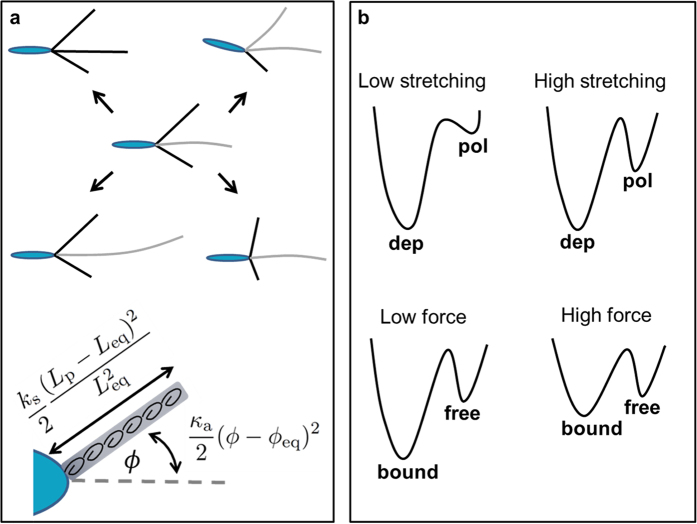
Kinetic Monte Carlo simulations. (**a)** Examples of processes that bacteria can undergo in a KMC simulation step (black line: surface bound pilus, grey line: free pilus). Below is a sketch of a pilus eminating from the cell pole with the visualization of the two elastic terms. (**b)** The energy landscapes illustrating how the switching rate from a depolymerizing (dep) to a polymerizing (pol) state depends on the stretching tension (top) and how the pulling force on the pilus increases the rate of release from the surface *K*_Rel_.

**Figure 3:**
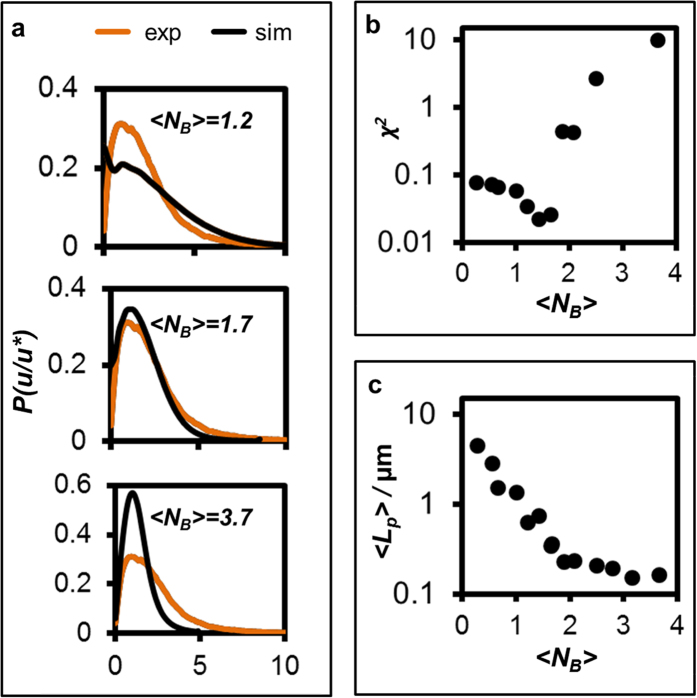
Slingshot bacteria preform two-legged crawling. (**a**) Scaled speed distribution *P*(*u*/u^*^) of slingshot bacteria from the experiments (orange) and simulations (black). **(b)** The discrepancy between the experiments and simulations, *χ*^2^, as a function of 〈*N*_B_〉. The best match between the simulations and the experiment is achieved when *χ*^2^ is minimal, i.e. 〈*N*_B_〉 ≈ 2. (**c**) The mean length of TFP in the simulation, 〈*L*_*p*_〉, as a function of 〈*N*_B_〉. When bound, TFP predominantly retract and do not elongate affecting their average length.

**Figure 4:**
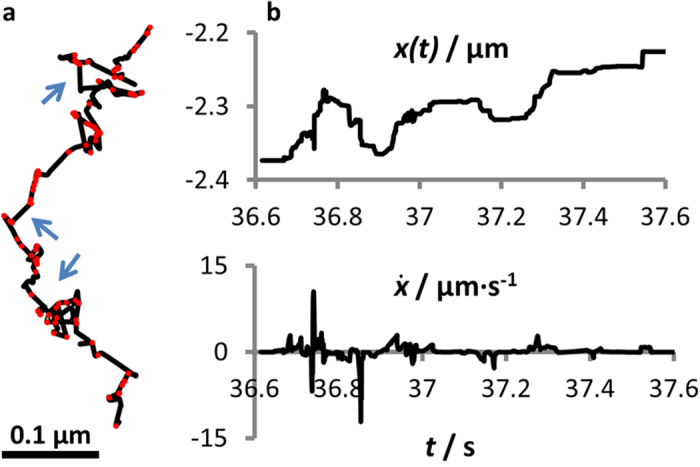
Slow and fast modes. (**a**) Part of a trajectory from computer simulations. Red dots indicate pilus-release events, blue arrows point on fast jumps, with above-threshold velocity of 0.07 *μ*m/s. Jumps always occur after a release, however not all the release event result in a jump. (**b**) Time evolution of the *x*-coordinate and its time derivative. The parameters used here are *K*_Rel_ = 130 s^−1^, *K*_Ad_ = 5 s^−1^, *k*_a_ = 10^−14^ and *ν* = *π*/8.

**Figure 5:**
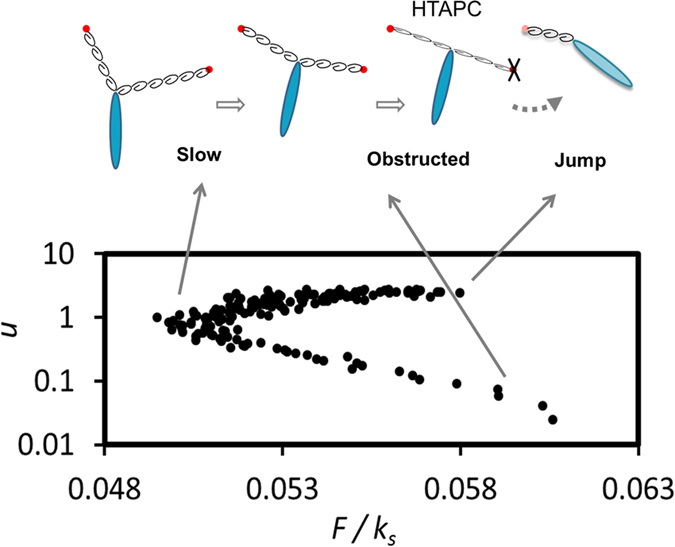
Tension-mediated pili cooperativity. The correlation of bacterial instantaneous crawling speed *u* and the tension in the filaments *F*/*k*_s_ for ‘two-legged crawling’ with rotationally flexible TFP. The TFP configuration in various parts of the diagram are illustrated above: If pili are not antiparallel, they can relax the tension by reorientation and the cell motion is slow and persistent. In the HTAPC the tension cannot be relaxed resulting in either obstructed tug-of-war configuration or a fast ‘sling shot’ jump if one of the pili is released. The parameter values are *K*_Rel_ = 130 s^−1^, *K*_Ad_ = 5 s^−1^ (corresponding to 〈*N*_B_〉 = 1.7), *k*_a_ = 10^−14^ and *ν* = *π*/8.

**Figure 6:**
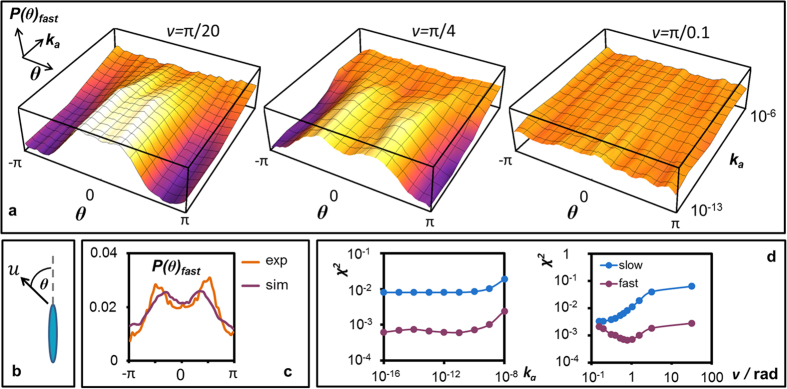
Sling-shot characterization. (**a**) Distribution *P*(*θ*) of the angular reorientation *θ* in the fast modes as a function of motor flexibility *k*_a_ for three values of variance *ν: π*/20, *π*/4, and 10*π*. The pronounced peaks at *π*/2 are only observed at intermediate *ν* and low *k*_a_. (**b**) definition of *θ*, the angle between cell axis and velocity. (**c**) Comparison of the distribution *P*(*θ*) observed in the experiments and in simulations with *ν* = *π*/4 and *k*_a_ = 10^−11^. (**d**) The discrepancy *χ*^2^ between the experimental observations and simulations in the speed distributions for the fast mode is shown at constant *ν* = *π*/4 as a function of the motor flexibility *k*_a_ (left), and at constant *k*_a_ = 10^−13^ as a function of the variance *ν* (right). In all of the plots *K*_Rel_ = 130 s^−1^ and *K*_Ad_ = 5 s^−1^, corresponding to 〈*N*_B_〉 ≈ 1.7.

**Figure 7:**
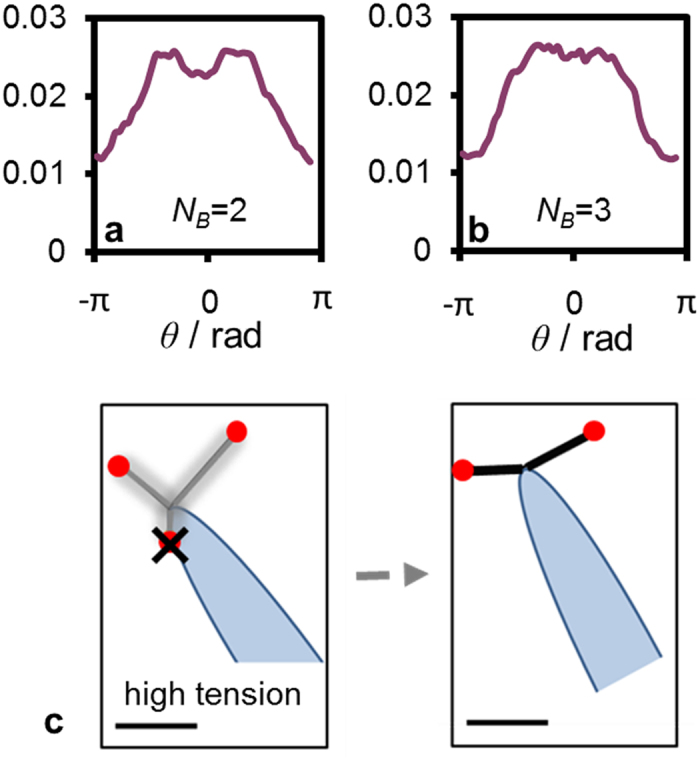
Sling-shot jumps result from release events with two bound pili. Frequency of the fast mode reorientation angles *θ* at optimal sling shot parameters *ν* = *π*/4 and *k*_a_ = 10^−11^: The events are separated into those involving two (**a**) or three (**b**) TFP - clearly demonstrating that the experimentally observed slingshots result from two-pili processes. (**c**): Simulation snapshot showing a configuration of high tension with three bound pili. In such cases the tension in the filaments cannot be relaxed by reorientation and thus prior to the release the TFP orientations are broadly distributed - in the depicted snapshot the release event resulted in a forward jump. The scale bar at the bottom is 0.2 *μ*m.

Clausen *et al*.^34^ showed that under low stretching force bound TFP predominantly retract, while increasing the force enhances the probability of TFP elongation. To model the switching dynamics between these two states, we assume an energy landscape at which the two modes are represented by minima separated by a barrier (Fig. 2b). The energy level corresponding to the retracting mode is lower than the elongation level and does not depend on the force, while the elongation level linearly decreases with the stretching force up to the stall force of the pilus motor, *F*_stall_ = 100 pN^2^,^3^. The transition rates, in agreement with experimental findings^34^, are assumed to be


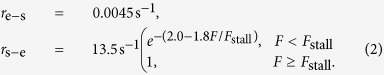


The probability for pilus release is assumed to increase with the force (see illustration in Fig. 2b). :


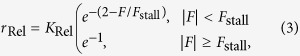


with *K*_Rel_ the release constant of the TFP from the surface. The adhesion rate, *r*_Ad_ = *K*_Ad_ is a force-independent parameter.

## Additional Information

**How to cite this article**: Brill-Karniely, Y. *et al*. Emergence of complex behavior in pili-based motility in early stages of *P. aeruginosa* surface adaptation. *Sci. Rep.* **7**, 45467; doi: 10.1038/srep45467 (2017).

**Publisher's note:** Springer Nature remains neutral with regard to jurisdictional claims in published maps and institutional affiliations.

## Supplementary Material

Supplementary Information

## References

[b1] Merz A. J., So M. & Sheetz M. P. Pilus retraction powers bacterial twitching motility. Nature 407, 98–102 (2000). 10993081 10.1038/35024105

[b2] Maier B. . Single pilus motor forces exceed 100 pN. Proc. Natl. Acad. Sci. USA 99, 16012–16017 (2002). 12446837 10.1073/pnas.242523299 PMC138556

[b3] Clausen M., Jakovljevic V., Sogaard-Andersen L. & Maier B. High-force generation is a conserved property of type IV pilus systems. J. Bacteriol. 191, 4633–4638 (2009). 19429611 10.1128/JB.00396-09 PMC2704717

[b4] Baker J. L., Biais N. & Tama F. Steered Molecular Dynamics Simulations of a Type IV Pilus Probe Initial Stages of a Force-Induced Conformational Transition. PLoS Comput. Biol. 9, e1003032 (2013). 23592974 10.1371/journal.pcbi.1003032 PMC3623709

[b5] Skerker J. M. & Berg H. C. Direct observation of extension and retraction of type IV pili. Proc. Natl. Acad. Sci. USA 98, 6901–6904 (2001). 11381130 10.1073/pnas.121171698 PMC34450

[b6] Bradley D. E. Function of Pseudomonas-Aeruginosa Pao Polar Pili - Twitching Motility. Can. J. Microbiol. 26, 146–154 (1980). 6105908 10.1139/m80-022

[b7] Henrichsen J. Twitching Motility. Annu. Rev. Microbiol. 37, 81–93 (1983). 6139059 10.1146/annurev.mi.37.100183.000501

[b8] Burrows L. L. Weapons of mass retraction. Mol. Microbiol. 57, 878–888 (2005). 16091031 10.1111/j.1365-2958.2005.04703.x

[b9] Kaiser D. Bacterial motility: How do pili pull? Curr. Biol. 10, R777–R780 (2000). 11084348 10.1016/s0960-9822(00)00764-8

[b10] Mattick J. S. Type IV pili and twitching motility. Annu. Rev. Microbiol. 56, 289–314 (2002). 12142488 10.1146/annurev.micro.56.012302.160938

[b11] Merz A. J. & Forest K. T. Bacterial surface motility: slime trails, grappling hooks and nozzles. Curr. Biol. 12, R297–R303 (2002). 11967173 10.1016/s0960-9822(02)00806-0

[b12] Lu S. . Nanoscale Pulling of Type IV Pili Reveals Their Flexibility and Adhesion to Surfaces over Extended Lengths of the Pili. Biophys. J. 108, 2865–2875 (2015). 26083926 10.1016/j.bpj.2015.05.016 PMC4472224

[b13] Strom M. S. & Lory S. Structure-function and biogenesis of the type IV pili. Annu. Rev. Microbiol. 47, 565–596 (1993). 7903032 10.1146/annurev.mi.47.100193.003025

[b14] Burrows L. L. Pseudomonas aeruginosa Twitching Motility: Type IV Pili in Action. Annu. Rev. Microbiol. 66, 493–520 (2012). 22746331 10.1146/annurev-micro-092611-150055

[b15] O’Toole G. A. & Kolter R. Flagellar and twitching motility are necessary for Pseudomonas aeruginosa biofilm development. Mol. Microbiol. 30, 295–304 (1998). 9791175 10.1046/j.1365-2958.1998.01062.x

[b16] Conrad J. C. . Flagella and Pili-Mediated Near-Surface Single-Cell Motility Mechanisms in P. aeruginosa. Biophys. J. 100, 1608–1616 (2011). 21463573 10.1016/j.bpj.2011.02.020 PMC3072661

[b17] Gibiansky M. L. . Bacteria Use Type IV Pili to Walk Upright and Detach from Surfaces. Science 330, 197–U50 (2010). 20929769 10.1126/science.1194238

[b18] Chiang P. & Burrows L. L. Biofilm formation by hyper-piliated mutants of Pseudomonas aeruginosa. J. Bacteriol. 185, 2374–2378 (2003). 12644510 10.1128/JB.185.7.2374-2378.2003 PMC151504

[b19] Klausen M. . Biofilm formation by Pseudomonas aeruginosa wild type, flagella and type IV pili mutants. Mol. Microbiol. 48, 1511–1524 (2003). 12791135 10.1046/j.1365-2958.2003.03525.x

[b20] Singh P. K., Parsek M. R., Greenberg E. P. & Welsh M. J. A component of innate immunity prevents bacterial biofilm development. Nature 417, 552–555 (2002). 12037568 10.1038/417552a

[b21] Semmler A. B., Whitchurch C. B. & Mattick J. S. A re-examination of twitching motility in Pseudomonas aeruginosa. Microbiology+ 145, 2863–2873 (1999). 10537208 10.1099/00221287-145-10-2863

[b22] Conrad J. C. Physics of bacterial near-surface motility using flagella and type IV pili: implications for biofilm formation. Res. Microbiol. 163, 619–629(2012). 23103335 10.1016/j.resmic.2012.10.016

[b23] Wang S., Parsek M. R., Wozniak D. J. & Ma L. Z. A spider web strategy of type IV pili-mediated migration to build a fibre-like Psl polysaccharide matrix in Pseudomonas aeruginosa biofilms. Environ. Microbiol. 15, 2238–2253 (3013). 23425591 10.1111/1462-2920.12095 PMC4466117

[b24] Persat A., Inclan Y. F., Engel J. N., Stone H. A. & Gitai Z. Type IV pili mechanochemically regulate virulence factors in Pseudomonas aeruginosa. Proc. Natl. Acad. Sci. USA 24, 7563–7568 (2015). 10.1073/pnas.1502025112 PMC4475988 26041805

[b25] Persat A. . The Mechanical World of Bacteria. Cell 161, 988–997 (2015). 26000479 10.1016/j.cell.2015.05.005 PMC4451180

[b26] Maier B. & Wong G. C. L. How Bacteria Use Type IV Pili Machinery on Surfaces. Trends in Microbiology 23, 775–788 (2015). 26497940 10.1016/j.tim.2015.09.002

[b27] Maier B. The bacterial type IV pilus system – a tunable molecular motor. Soft Matter 9, 5667–5671 (2013).

[b28] Zhang R., Ni L., Jin Z., Li J. & Jin F. Bacteria slingshot more on soft surfaces. Nature Communications 5, 5541 (2014). 10.1038/ncomms6541 PMC4263163 25412641

[b29] Meel C., Kouzel N., Oldewurtel E. R. & Maier B. Three-Dimensional Obstacles for Bacterial Surface Motility. Small 8, 530–534 (2012). 22183854 10.1002/smll.201101362

[b30] Zaburdaev V. . Uncovering the Mechanism of Trapping and Cell Orientation during Neisseria gonorrhoeae Twitching Motility. Biophysical Journal 107, 1523–1531 (2014). 25296304 10.1016/j.bpj.2014.07.061 PMC4190650

[b31] Holz C. . Multiple Pilus Motors Cooperate for Persistent Bacterial Movement in Two Dimensions. Phys. Rev. Lett. 104, 178104 (2010). 20482147 10.1103/PhysRevLett.104.178104

[b32] Kurre R. & Maier B. Oxygen Depletion Triggers Switching between Discrete Speed Modes of Gonococcal Type IV Pili. Biophysical Journal 102, 2556–2563 (2012). 22713571 10.1016/j.bpj.2012.04.020 PMC3368137

[b33] Marathe R. . Bacterial twitching motility is coordinated by a two-dimensional tug-of-war with directional memory. Nature Communications 5, 3759 (2014). 10.1038/ncomms4759 24806757

[b34] Clausen M., Koomey M. & Maier B. Dynamics of Type IV Pili Is Controlled by Switching Between Multiple States. Biophys. J. 96, 1169–1177 (2009). 19186152 10.1016/j.bpj.2008.10.017 PMC2716576

[b35] Kurre R., Kouzel N., Ramakrishnan K., Oldewurtel E. R. & Maier B. Speed switching of gonococcal surface motility correlates with proton motive force. PLoS One 8, e67718 (2013). 23826337 10.1371/journal.pone.0067718 PMC3691265

[b36] Zhao K. . Psl trails guide exploration and microcolony formation in Pseudomonas aeruginosa biofilms. Nature 497, 388–391 (2013). 23657259 10.1038/nature12155 PMC4109411

[b37] Jin F., Conrad J. C., Gibiansky M. L. & Wong G. C. L. Bacteria use type-IV pili to slingshot on surfaces. Proc. Natl. Acad. Sci. USA 108, 12617–12622 (2011). 21768344 10.1073/pnas.1105073108 PMC3150923

[b38] OToole G. A. & Wong G. C. L. Sensational Biofilms: Surface Sensing in Bacteria. Current Opinion in Microbiology 30, 139–146 (2016). 26968016 10.1016/j.mib.2016.02.004 PMC4843124

[b39] Luo Y. . A Hierarchical Cascade of Second Messengers Regulates Pseudomonas aeruginosa Surface Behaviors. mBio 6, 1–11 (2015). 10.1128/mBio.02456-14 PMC4324313 25626906

[b40] Weiss R. L. The structure and occurrence of pili (fimbriae). on Pseudomonas aeruginosa. J. Gen. Microbiol. 67, 135–143 (1971). 4108909 10.1099/00221287-67-2-135

[b41] Rheinlaender J., Grabner A., Ott L., Burkovski A. & Schaffer T. E. Contour and persistence length of Corynebacterium diphtheriae pili by atomic force microscopy. Eur. Biophys. J. 41, 561–570 (2012). 22588485 10.1007/s00249-012-0818-4

[b42] Nudleman E., Wall D. & Kaiser D. Polar assembly of the type IV pilus secretin in Myxococcus xanthus. Mol. Microbiol. 60, 16–29 (2006). 16556217 10.1111/j.1365-2958.2006.05095.x

[b43] Maier B., Koomey M. & Sheetz M. P. A force-dependent switch reverses type IV pilus retraction. Proc. Natl. Acad. Sci. USA 101, 10961–10966 (2004). 15256598 10.1073/pnas.0402305101 PMC503726

[b44] Gillespie D. T. Exact Stochastic Simulation of Coupled Chemical-Reactions. J. Phys. Chem. 81, 2340–2361 (1977).

[b45] Chang Y.-W. . Architecture of the type IVa pilus machine. Science 351, 1165 (2016). 10.1126/science.aad2001 PMC5929464 26965631

[b46] Son K., Guasto J. S. & Stocker R. Bacteria can exploit a flagellar buckling instability to change direction. Nature Physics 9, 494–498 (2013).

[b47] Berg H. C. Turning failure into function. Nature Physics 9, 460–461 (2013).

[b48] Deziel E., Comeau Y. & Villemur R. Initiation of Biofilm Formation by Pseudomonas aeruginosa 57RP Correlates with Emergence of Hyperpiliated and Highly Adherent Phenotypic Variants Deficient in Swimming, Swarming, and Twitching Motilities. J. Bacteriol. 183, 1195–1204 (2001). 11157931 10.1128/JB.183.4.1195-1204.2001 PMC94992

