# Supplementary material for: Emergence of complex behavior in pili-based motility in early stages of P. aeruginosa surface adaptation
Source: Sci Rep. 2017 Apr 10;7:45467. doi: 10.1038/srep45467 (PMC5385500; doi:10.1038/srep45467)
Supplement: Supplementary Information [file srep45467-s1.pdf]

Emergence of complex behavior in pili-based motility in early stages of *P. aeruginosa* surface adaptation

Supporting information

Yifat Brill-Karniely[1;2], Fan Jin[3;4], Gerard Wong[4], Daan Frenkel[1], and Jure Dobnikar[1;5]

[1] Department of Chemistry, University of Cambridge, Lensfield Road, CB2 1EW, Cambridge, UK

[2] Institute for Drug Research, Faculty of Medicine,

The Hebrew University of Jerusalem, Jerusalem, Israel, 91120

[3] Hefei National Laboratory for Physical Sciences at Microscale,

Department of Polymer Science and Engineering, CAS Key Laboratory of Soft Matter Chemistry,

University of Science and Technology of China, Hefei, P. R. China 230026

[4] Bioengineering Department, Chemistry and Biochemistry Department,

California Nano Systems Institute, University of California, Los Angeles, CA 90095, USA

[5] Institute of Physics, Chinese Academy of Sciences, 100190 Beijing, P. R. China

Supplementary Figures

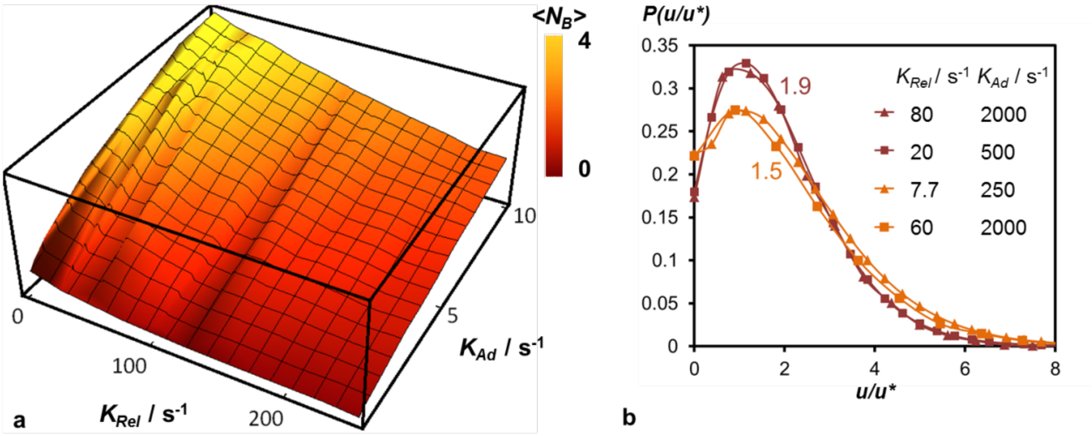

Supplementary Figure 1. (a) The average number of bound TFP is determined by a feedback mechanism resulting from the enhanced probability of shorter pili to detach. $<N_B>$ is plotted as a function of the rate constants for surface adhesion and release. $<N_B>$ is measured in the simulations given K_{Rel} and K_{Ad}. In general, the higher is the on - rate, and the lower is the off - rate there are more bound pili. However, complexity arises from the dependence of the release rate on the length of the bound TFP. The shorter bound TFP are the higher is the probability for their release as they can develop higher tension. Moreover, pili that retract to a vanishing length detach from the surface. In other words, shorter TFP have higher probability to detach and to reduce $<N_B>$. This leads to a feedback mechanism since the increase in the number of free TFP, in turn, results in higher length of the pili as they have more opportunity to elongate (see **Figure 3c,** $<Lp>$ vs $<N_B>$). Longer pili then have lower probability for detachment after binding the surface. This oscillatory feedback behavior is seen in the ruffled shape of $<N_B>$ surface plot when varying K_{Rel}, as K_{Rel} controls the life time of bound TFP, namely the duration of retraction and the subsequent increase in the release probability. **(b)** Scaled bacteria velocity distribution depends on the average number of bound pili and not on the specific values of the adhesion / release rates. Presented are four examples of distributions that correspond to different sets of the rate constants, with $<N_B>\approx1.9$ or 1.5. The distributions for couples with the same $<N_B>$ are similar - in spite of the differences in the rate constants.

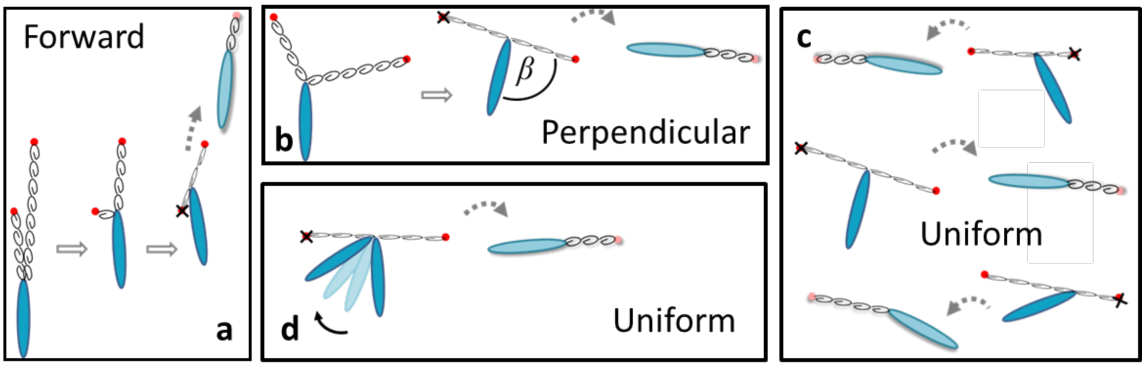

Supplementary Figure 2. The reorientation of bacteria upon a “jump” event is controlled by the value of the angle between the cell and the bound TFP axis, at the moment of pilus release in HTAPC. As explained in the main text, in the case of extremely flexible anchors, three modes of jumps were observed, depending on the equilibrium distribution of TFP: **(a)** forward (e.g. v=π/20 in Figure 6A), **(b)** perpendicular (see v=π/4 in Figure 6A) and **(c)** uniform (v=π/0.1 in Figure 6A). **(d)** If TFP anchors are not sufficiently flexible (k_a≥10^{-9}) bacteria rotate around the TFP axis in HTAPC, leading to a uniform distribution of jump angles upon a pilus release. In all the illustrations, pili at HTAPC are marked by thinner springs for clarity.

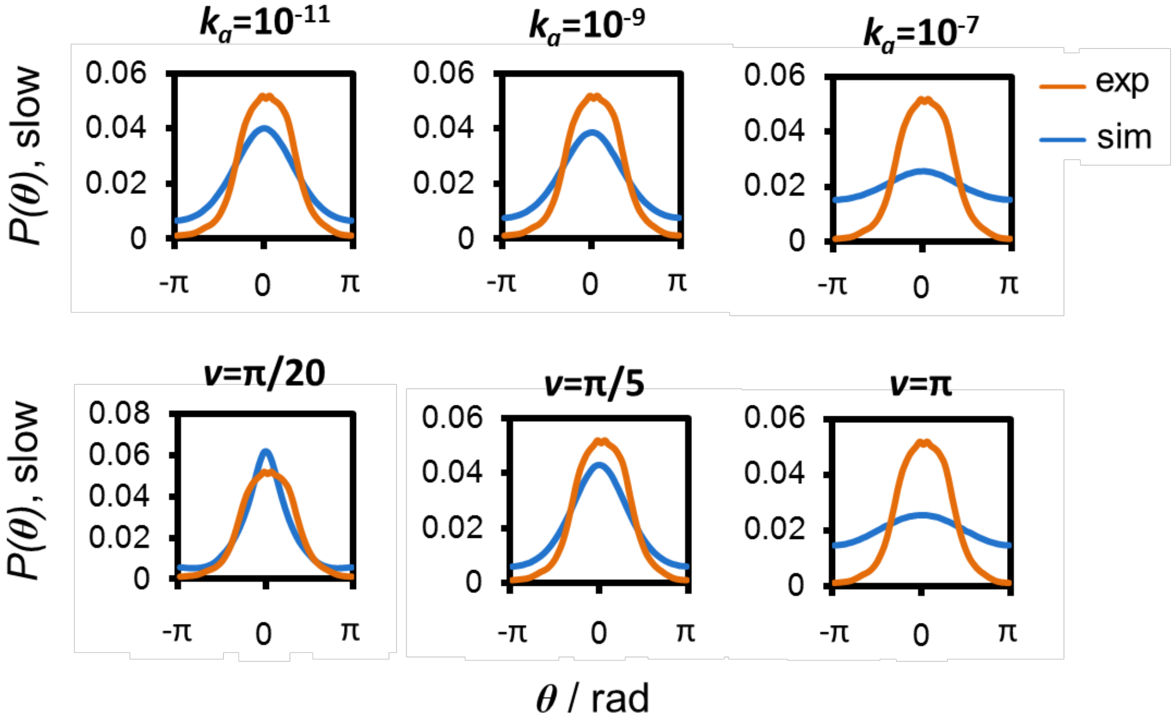

Supplementary Figure 3. TFP anchor rigidity and dispersion of equilibrium angles control the persistence of the slow mode. Shown are results from the experiments (orange) and from the simulations with varying k_a and *v* (blue). The directionality of the motion smears when raising anchor rigidity and also when widening the angular distribution. Elevated anchor flexibility leads to higher dispersion of free TFP angles, which limits the directionality of the motion. Interestingly, the best match between the experiments and simulations for the slow mode is achieved for moderate values of *v* and extremely flexible anchors, in perfect correlation with the optimal parameters for the fast mode (see discrepancy curves in Figure 6). Parameters used here are K_{Rel}=130 s^{-1}, K_{Ad}=5 s^{-1}, *v*=π/8 (upper panel) and k_a=10^{-13} (lower panel).

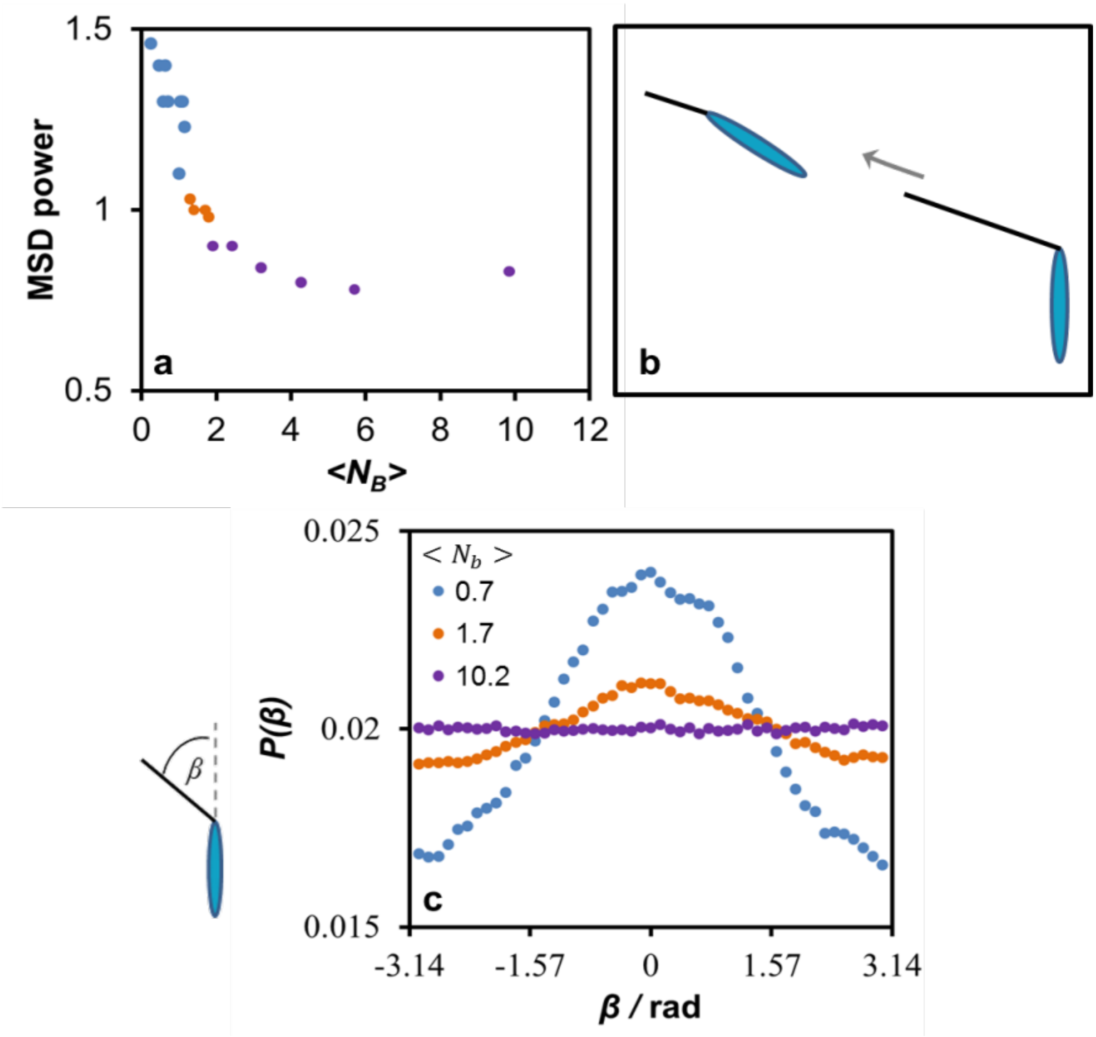

Supplementary Figure 4. Bacteria with *uniform* distribution of new pili angles can form different motion types, depending on the average number of bound pili. **(a)** A *trapped* motion (purple, MSD power<1) occurs when multiple pili obstruct each other; a *diffusive* motion (orange) is dominated by single pili retracting in arbitrary directions; and for $< N_B >$ below 1.3 pili reorient parallel to bacterium axis **(b)** resulting in *a localized directional* motion (blue circles in **a**). In general, the lower is $< N_B >$ the more prominent is the narrowing of pili angular distribution **(c)**.

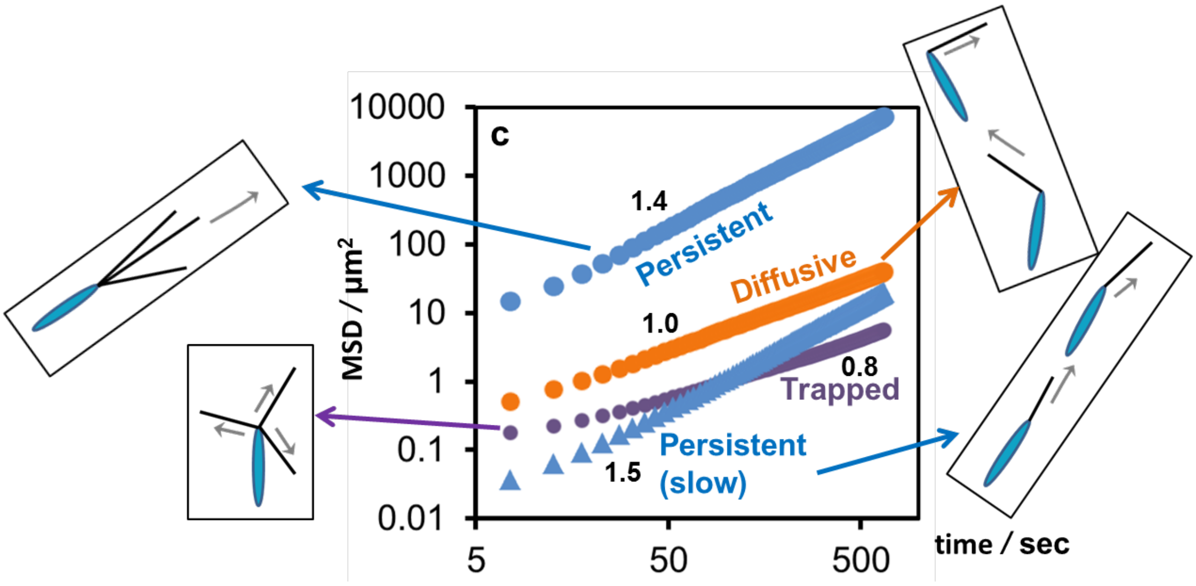

Supplementary Figure 5. Demonstration of MSD plots for the motion phases observed in Figure 1 of the main text. MSD power values are presented near the plots. For each mode, a typical bacterium illustration is shown.

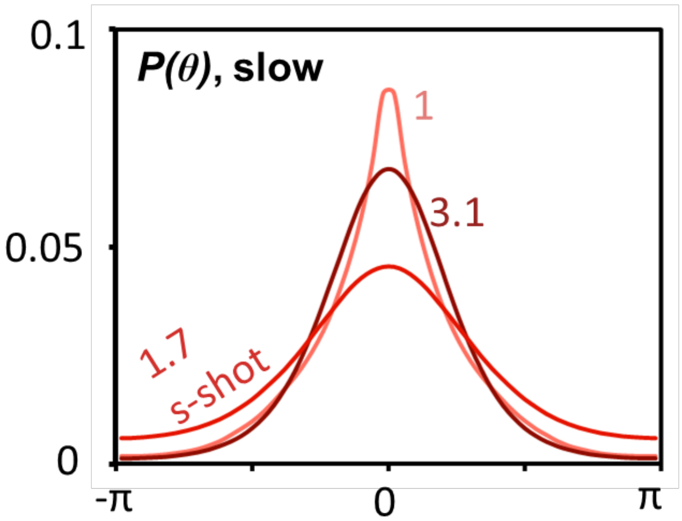

Supplementary Figure 6. Due to the tendency to reach HTAPC, in the slingshot mode TFP angular distribution widens. Shown are plots of $P(\theta)$ of the slow mode, which indicate of TFP angular dispersity. The thickness of the distribution changes in a non-monotonic manner with $\langle N_B \rangle$. Note the wide spread in slingshot conditions. Also, for $\langle N_B \rangle < 1.5$, the TFP angular spread becomes narrow (due to the reasons explained in Figure S4). In other words the angular distribution of TFP in slingshot bacteria ($\langle N_B \rangle = 1.7$) is wider than for lower or higher numbers of bound pili. In all the plots $k_a = 10^{-13}$ and $v = \pi/6$.

Supplementary Tables

Supplementary Table 1: MSD slopes and the parameters used in **Fig. 1.**

**Mode**	$< N_B >$	v	**MSD power**	K_{Ad} /s^{-1}	K_{Rel} /s^{-1}
Trapped	9.8	uni	0.83	200	10
	5.7	uni	0.78	20	1
	4.3	uni	0.80	10	1
	3.2	uni	0.84	5	10
	2.4	uni	0.90	5	50
	1.9	uni	0.90	5	100
Diffusive	1.8	uni	0.98	1.4	1
	1.7	uni	1.0	5	130
	1.4	uni	1.0	4	130
	1.3	uni	1.03	3.75	130
Localized directional	1.1	uni	1.23	11	500
	1.1	uni	1.3	10	500
	1.0	uni	1.3	80	5000
	1.0	uni	1.1	3	130
	0.7	uni	1.3	2	130
	0.6	uni	1.3	50	5000
	0.6	uni	1.4	5	500
	0.5	uni	1.4	1	130
	0.25	uni	1.46	1	200
Rapid exploration	10.2	π/2	1.65	200	0.0001
	7.2	π/2	1.44	50	1
	5.7	π/2	1.3	20	1
	4.3	π/2	1.2	10	1
	1.7	π/2	1.35	5	130
	0.6	π/2	1.67	5	500
	10.2	π/3	1.67	200	0.0001
	7.2	π/3	1.55	50	1
	5.7	π/3	1.3	20	1
	4.3	π/3	1.2	10	1
	1.7	π/3	1.38	5	130
	0.6	π/3	1.72	5	500
	10.2	π/8	1.68	200	0.0001
	7.2	π/8	1.6	50	1
	5.7	π/8	1.43	20	1
	4.3	π/8	1.29	10	1
	1.7	π/8	1.43	5	130
	0.6	π/8	1.77	5	500
